# 2D alpha-shapes to quantify retinal microvasculature morphology and their application to proliferative diabetic retinopathy characterisation in fundus photographs

**DOI:** 10.1038/s41598-021-02329-5

**Published:** 2021-11-24

**Authors:** Emma Pead, Ylenia Giarratano, Andrew J. Tatham, Miguel O. Bernabeu, Baljean Dhillon, Emanuele Trucco, Tom MacGillivray

**Affiliations:** 1 VAMPIRE Project, Scottish Collaborative Optometry-Ophthalmology Network e-Research (SCONe), Centre for Clinical Brain Sciences, The University of Edinburgh, Edinburgh, UK; 2 Centre for Medical Informatics, Usher Institute, The University of Edinburgh, Edinburgh, UK; 3 Princess Alexandra Eye Pavilion, NHS Lothian, Edinburgh, UK; 4 VAMPIRE Project, CVIP, Computing (SSE), University of Dundee, Dundee, UK

**Keywords:** Predictive markers, Retinal diseases, Predictive markers, Image processing

## Abstract

The use of 2D alpha-shapes (α-shapes) to quantify morphological features of the retinal microvasculature could lead to imaging biomarkers for proliferative diabetic retinopathy (PDR). We tested our approach using the MESSIDOR dataset that consists of colour fundus photographs from 547 healthy individuals, 149 with mild diabetic retinopathy (DR), 239 with moderate DR, 199 pre-PDR and 53 PDR. The skeleton (centrelines) of the automatically segmented retinal vasculature was represented as an α-shape and the proposed parameters, complexity (${Op\alpha }_{min}$), spread (*OpA*), global shape (VS) and presence of abnormal angiogenesis (*Grad*_*α*_) were computed. In cross-sectional analysis, individuals with PDR had a lower ${Op\alpha }_{min}$, OpA and *Grad*_*α*_ indicating a vasculature that is more complex, less spread (i.e. dense) and the presence of numerous small vessels. The results show that α-shape parameters characterise vascular abnormalities predictive of PDR (AUC 0.73; 95% CI [0.73 0.74]) and have therefore potential to reveal changes in retinal microvascular morphology.

## Introduction

DR is a leading cause of preventable blindness and a major complication of diabetes mellitus^1^. DR is classified as non-proliferative, if there are vascular abnormalities such as microaneurysms, or PDR if damage to the retina has become sufficient to stimulate growth of abnormal retinal blood vessels (neovascularisation). New vessels are weak and have a high risk of causing irreversible visual loss through leakage, bleeding, or retinal detachment. Identifying patients at high risk of progression to PDR is critical for providing timely sight-preserving treatment. PDR risk assessment is conducted through manual grading of fundus photographs obtained through nationwide diabetic retinopathy screening programmes^2^. The identification of parameters obtainable automatically for quantification of pathological retinal microvascular remodelling, including the presence of neovascularisation, may permit early identification of PDR and improve patient outcomes.

Retinal vasculature morphology can be objectively quantified from fundus images through automated detection of retinal blood vessels and subsequent analysis of their geometric properties, including width, tortuosity, branching angles, and branching complexity via calculation of the fractal dimension (FD)^3^. FD is a unitless measure of the complexity of a pattern, describing how a self-similar pattern fills the space in which it is contained^4,5^. FD has previously been investigated as a imaging biomarker in neurodegenerative diseases and in conditions with abnormal angiogenesis such as diabetic retinopathy (DR)^6–12^.

For PDR, conflicting findings with FD have been reported. A higher FD, indicating increased vascular complexity, has been reported in PDR than in healthy controls^13,14^, whilst a lower FD, indicating a decreased vascular complexity, has been reported when comparing PDR to patients with Type I diabetes^15^ and when comparing PDR to healthy controls^16^. Huang et al*.*^17^ reported no evidence of a significant difference in FD between DR grades and Orlando et al. reported a higher FD in PDR when compared to other DR grades^13^. Such variation in previously reported FD have been attributed to lack of standardisation in FD calculation^17,18^. FD and it’s calculation can be affected by: (1) the accuracy of vessel segmentation; (2) skeletonization, locating the centreline of each vessel; (3) camera resolution and image quality, which directly affects (1) and (2)^17^; (4) the FD algorithm and the choice of its parameters, such as the random sampling of points on the vessel map; and (5) the computational cost^19^, which may be excessive in a clinical setting.

In this study, we explore an alternative method of quantifying the global morphology of the retinal vasculature, using α-shapes. The α-shape is a well described geometric concept used to represent the boundary of a shape in 2D or 3D^20^. It has been applied previously to molecular shape characterisation^21^, brain tumour measurement in magnetic resonance imaging (MRI)^22^, cosmic web topology^23^, surface reconstruction^24^ and predicting species distributions^25^. Unlike FD, α-shapes are independent from sampling space, do not make assumptions of self-similarity, are computationally inexpensive and unaffected by parameter choice as no random sampling is conducted and there is only one α-shape for a point set^20^. We applied α-shapes to the task of characterising PDR, with a focus on deriving objective measurements that have a clinically interpretable output.

## Methods

### Materials and data

MESSIDOR is a publicly available dataset commonly used to evaluate performance of automatic DR detection systems and consists of 1187 fundus images acquired by a 45° FOV Topcon TRC NW6 non-mydriatic camera^26^. Of these 547 are grade 0 (no DR), 149 grade 1 (mild DR), 239 grade 2 (moderate), and 252 grade 3 (severe DR, consisting of pre-proliferative and PDR cases). Images are stored in TIF format and have three sizes: 1140 × 960 pixels, 2240 × 1488 pixels and 2304 × 1536 pixels. Segmentations and PDR image labels from Orlando et al.^13^ were accessed at https://github.com/ignaciorlando/fundus-fractal-analysis. In^13^, the authors standardised all images to 909 × 909 pixels to approximately match the calibre of the vessels appearing in images in the Digital Retinal Image for Vessel Extraction (DRIVE) dataset^27^. Images were then segmented using a fully connected conditional random field model^28^. Vessel skeletonization was performed using an iterative thinning algorithm that extracts the centre line of the vessels (1-pixel wide; curved lines), resulting in a vessel skeleton^29^. PDR labels were obtained by relabelling of DR grade 3 by two ophthalmologists whereby 53 images of grade 3 were assigned to the PDR grade (with neovessels)^13^. It should be noted that the relative number of images per DR group differ from^13^ due to the corrections outlined on the MESSIDOR project website which involved removal of 13 duplicate images and corrections of DR grades. We calculated FD using a multifractal approach and a generalised sandbox method, reported elsewhere^5^.

For assessing accuracy of α-shape descriptors and FD we used the publicly available high resolution fundus (HRF) dataset (available here: High-Resolution Fundus (HRF) Image Database (fau.de)). The HRF dataset contains 15 images of healthy people and 15 images of people with diabetic retinopathy (DR grades for these images are unavailable) with manual annotations of blood vessels. Images are 3504 × 2336 stored in JPEG format. Images were standardised to 1525 × 1017 pixels to approximately match the calibre of the DRIVE dataset. Images were segmented using the same procedure as the MESSIDOR dataset^13^.

### 2D α-shapes

**Figure 1:**
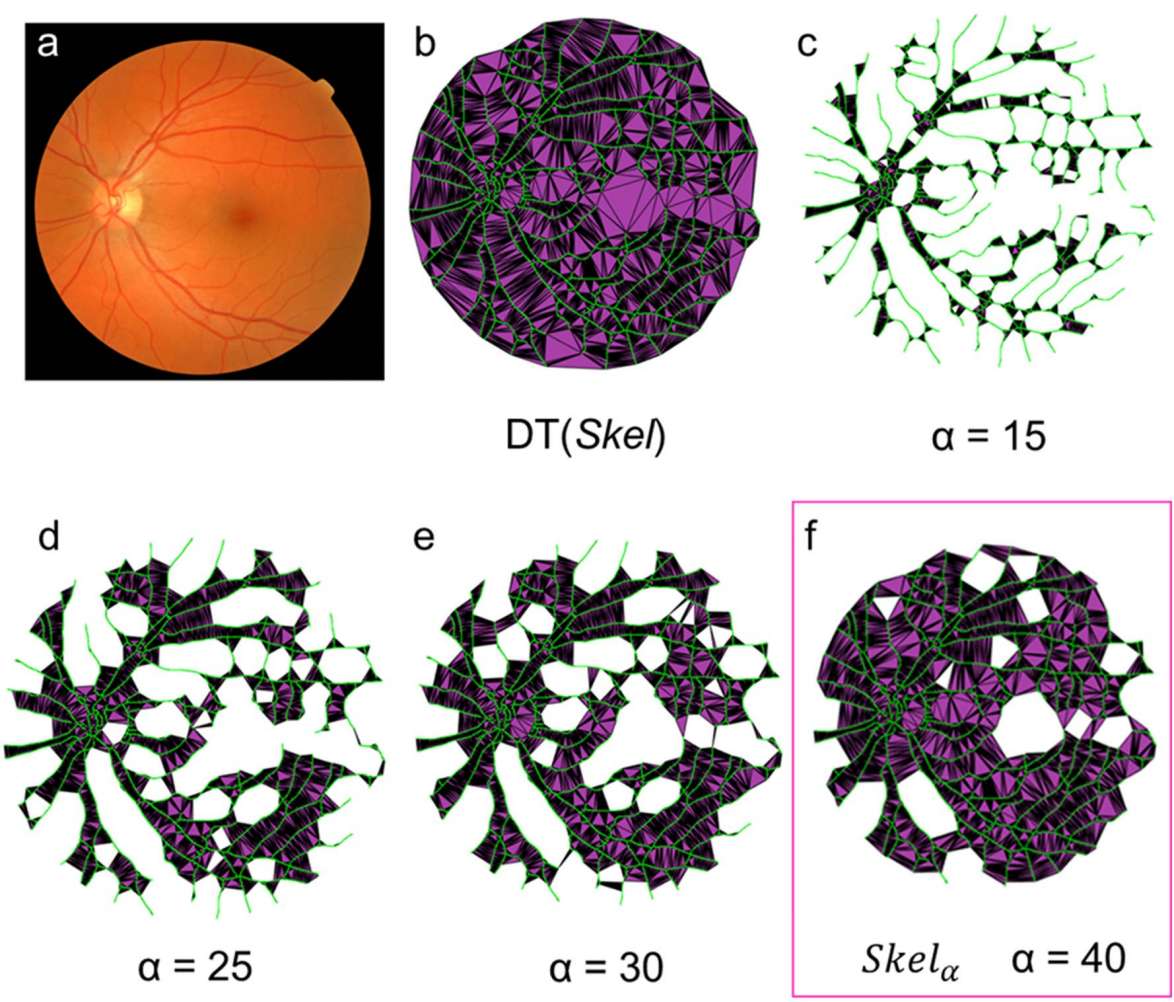
Representing the retinal microvasculature as a 2D alpha-shape. Controlling the level of refinement (α) from the vessel skeleton (green) of a colour fundus photograph **(a)** in the MESSIDOR dataset (Grade 0). (**b)** $\mathrm{DT}\left(\mathrm{Skel}\right)$ encompasses all points of the skeleton. **(c–e)** As α is increased more of the simplices in $\mathrm{DT}\left(\mathrm{Skel}\right)$ are included into the α-shape (purple), **(f)** until there is a minimum value of α that encompasses all points of the vasculature giving a single region and a representation of the vessel shape.

First, the retinal vasculature from a colour fundus photograph is skeletonised to obtain a map of centrelines of the blood vessels. Then a Delaunay Triangulation ($DT$) is computed from the points $S$ of the skeleton ($Skel$) (Fig. 1a,b). The α-shape can be considered as a subset of $DT$, whereby *Delaunay simplices—*i.e., triangles, edges, vertices—are removed to *refine* the boundary of the shape. Refinement is achieved by rolling a disc with radius α along each point in $S$, with an arbitrary starting point. If the next point touches the disc—i.e. the two points lie on the boundary of the disk with radius α and contains no other point in $S$—this becomes the new pivoting point and the corresponding edge of $DT$ is *collected*. All the *Delaunay simplices* that meet this criterion forms the α-shape. For α = 15 (pixels) (Fig. 1c), few points meet this criterion and forms an α-shape that includes a small number of $DT$ edges. As α is increased, more and more edges of $DT$ are *collected* (Fig. 1d,e). Eventually, there is a minimum value of α that encompasses all points of the vasculature giving a single region and a representation of the vessel shape (Fig. 1f). Finally, very large α equals $DT$ as all edges are eventually included and the shape is not refined. We considered the optimum α-shape of the retinal vasculature $({Skel}_{\alpha })$ to be the minimum value of α that encompassed all points in the skeleton giving only a single region (Fig. 1f).

### Computing 2D α-shape descriptors

For each image in the dataset the α-shape was computed (*alphaShape* function, Matlab r2019b, Mathworks, USA) applied to the vessel skeleton. We define complexity as the property of a vessel skeleton being composed of many parts (i.e., many branches and vessel paths); a complex vasculature would require more refinement (i.e., smaller α) than a less complex vasculature. Therefore, we extracted the minimum value of α that encompassed all points in the skeleton giving only a single region, termed ${Op\alpha }_{min}$, as a descriptor of the complexity of the vascular network. However, used alone ${Op\alpha }_{min}$ would not completely describe shape complexity, as a vessel skeleton may have a small ${Op\alpha }_{min}$ due to vessels being close together rather than complex with lots of branches. To model how the vessels are spread around the retina, the area of the optimum α-shape (in pixels), *OpA,* was computed. Using complexity and spread, we can define vessel shape (VS),

1 $$VS=\frac{OpA}{{Op\alpha }_{min}}$$

**Figure 2:**
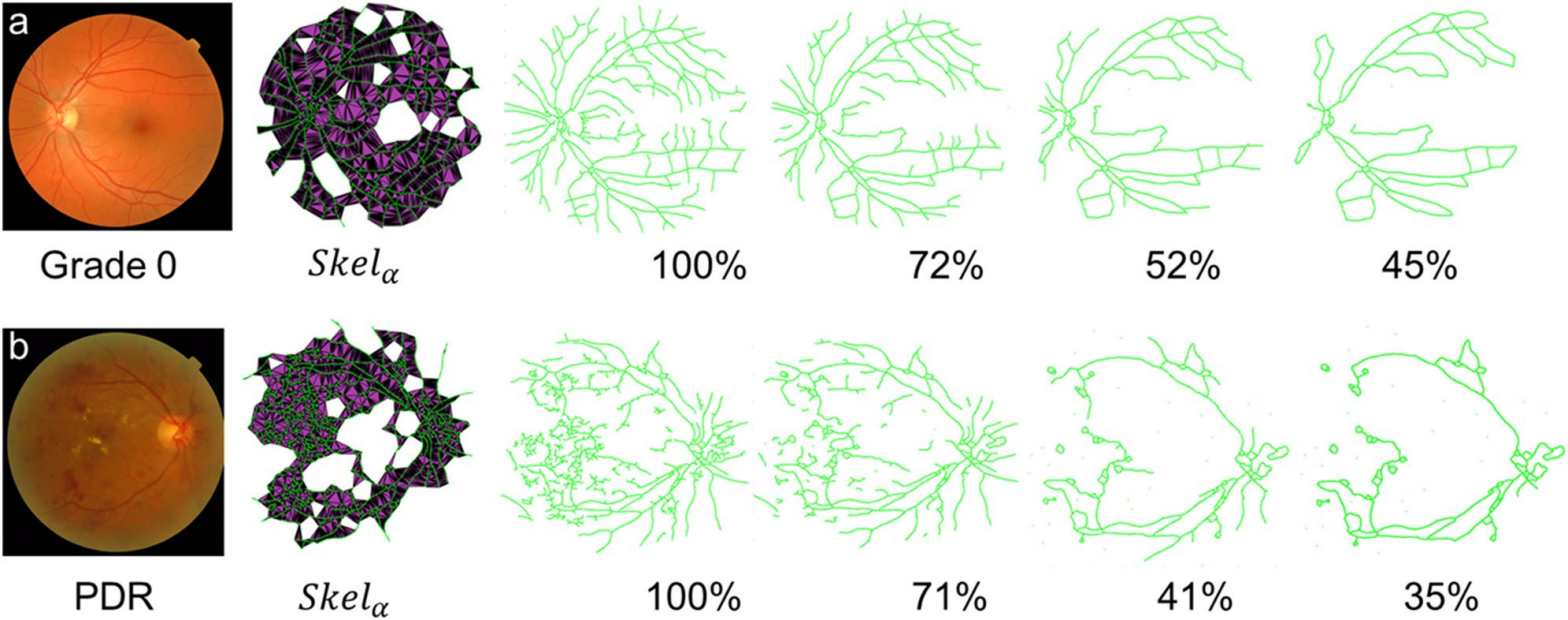
Example of vessel degradation using incremental morphological erosion. (**a)** Fundus image from a healthy participant (Grade 0, no PDR) and ${\mathrm{Skel}}_{\mathrm{\alpha }}$ overlaid onto the skeleton obtained from ^13^. The skeleton contains erroneous segmentations that are iteratively removed resulting in a skeleton 45% of the original skeleton. (**b)** Fundus image from PDR case and ${\mathrm{Skel}}_{\mathrm{\alpha }}$ overlaid onto the skeleton obtained from ^13^. Image is eroded to 35% of the original skeleton removing many of the minor blood vessels.

**Figure 3:**
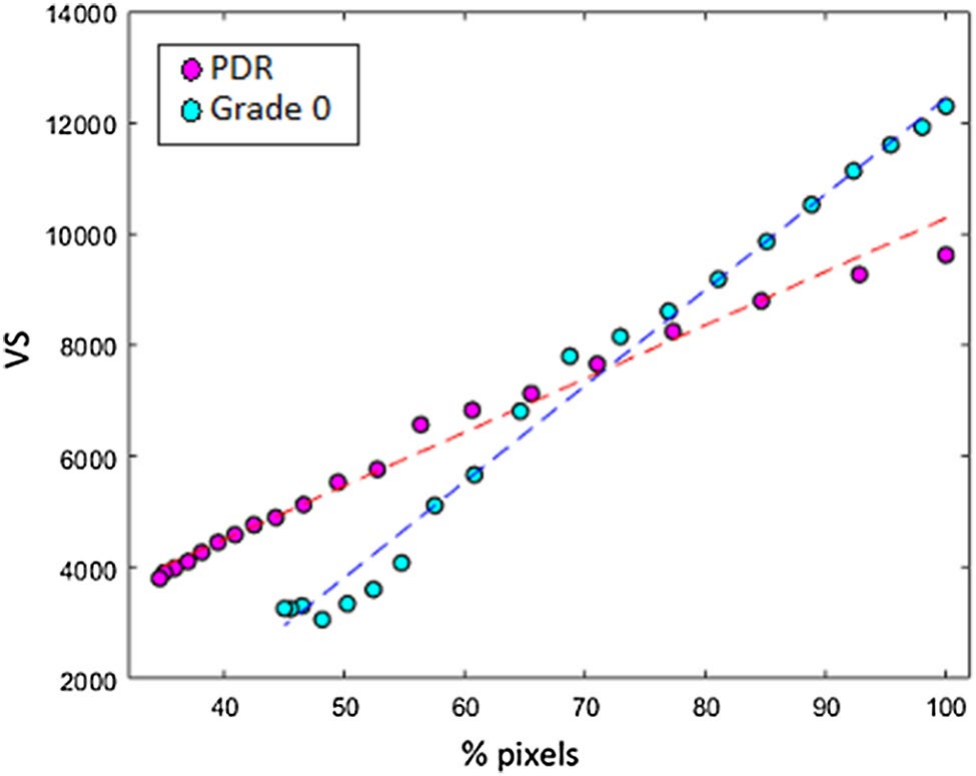
Evaluating the sensitivity of $\mathrm{VS}$ against changes in skeleton shape. Incremental removal of pixels (expressed as a percentage of the number of pixels of the original skeleton) against the candidate VS parameter for images in Fig. 2. As the skeleton is degraded the VS parameter also decreases, indicating that it is modelling change in vascular properties.

We evaluated the sensitivity of $VS$ against changes in skeleton shape by incremental morphological erosion of vessel skeletons. Each skeleton was eroded to approximately 30% of the original size (using the Matlab function *bwmorph* with parameter ‘*spur*’) to remove the ends of the vessels (20 iterations)^29^ (Fig. 2). A linear trend was observed and quantified by calculating the gradient of the robust regression line that fits the percentage of pixels in each erosion against $VS$, termed ${Grad}_{\alpha }$, to give an additional descriptor of vessel shape (Fig. 3). Intuitively, if the vasculature consists of many small branches, $VS$ will decrease at a lower rate. This is because the main vessels that create the overall shape will be preserved in early iterations of skeleton erosion. Likewise, if the vasculature consists of fewer small branches,$VS$ will decrease at a higher rate as the main vessels are eroded in early iterations. We therefore hypothesised that ${Grad}_{\alpha }$ could suggest the level of fragmentation of the vasculature, hence the presence of many small vessels typical of neovascularisation.

### Experiments and statistical analysis

Three experiments were conducted to address the following aims: (1) to assess the level of significance of the differences between all grades of DR (grades 1 to 3 and PDR); (2) evaluate the contribution of each α-shape descriptor and FD as features for PDR classification; and (3) assess the accuracy of α-shape descriptors and FD values.

Statistical analysis was conducted in R (version 3.6.0) and results were considered statistically significant at the significance level 0.05. In experiment (1), all groups (Grade 0 to 3 and PDR) were compared using Kruskall-Wallis test and p-values adjusted for multiple comparisons using Benjamani-Hochberg (data was not normally distributed with homogenous variance; Levene test, p > 0.05). Dunn post-hoc analysis was performed to assess which independent variable differed and p-values corrected for multiple comparisons using Bonferroni.

In experiment (2), α-shape descriptors as features for PDR characterisation were modelled using regularised logistic regression by grouping the data into PDR vs all other DR grades (grades 0 to 3). This was to identify features that could be used to distinguish neovascularisation (a defining feature of PDR) compared to other DR grades. Implementation of regularised logistic regression was performed using the R package *glmnet* (version 3.6.0) that performs model estimation and feature selection simultaneously^30,31^. We experimented with different strengths of regularisation (*λ*) and a choice of regularisation ($\mathrm{\alpha }\in [\mathrm{0,1}]$). Lasso regularisation (L_1_ regularisation; α = 1), ridge regularisation (L_2_ regularisation; α = 0) and a mixture of the two, elastic-net (α = 0.5). Lasso tends to discard correlated variables and select a single feature; ridge tends to equally weight features and shrink coefficients towards each other and elastic-net will exclude groups of highly corelated variables. As the α-shape descriptors are expected to be highly correlated, we applied lasso and elastic-net to perform feature selection.

Given that PDR cases consisted of 4.4% of the dataset and different sampling of the training data will give rise to different features being selected, we performed bootstrap analysis. A total of 500 bootstrap trials was performed and for each bootstrap trial the data was divided to keep proportions of classes equal (40 grade 0–3; 40 PDR) and model fitting was performed using fivefold cross-validation. Area Under the Curve (AUC) was used to assess model performance in each fold where the average AUC over all bootstrap trials is reported. fivefold cross validation simultaneously selects a value of *λ* where the highest AUC on the validation set is achieved (*λ*_min_) and the highest AUC on the validation set within one standard error of *λ*_min_ (*λ*_1SE_). Feature importance was assessed by counting the number of times each coefficient reached a non-zero weight in each bootstrap. As *λ*_min_ and *λ*_1SE_ will return different numbers of features (i.e. *λ*_1SE_ will contain less features) we assessed feature importance for both values of *λ*^32^.

In experiment (3), the Dice similarity coefficient (DSC) was used to evaluate performance of the automatic segmentation. To investigate variation in α-shape descriptors and FD measures obtained from the manual and automatic segmentation we use relative error (manual annotation as reference). Relative error is obtained by |AV-MV|/AV, where AV is the value of α-shape descriptors or FD obtained from the manual segmentation and MV is the value of α-shape descriptors or FD obtained from the automatic segmentation. To test whether measures obtained manually and automatically were correlated we used Pearson correlation coefficient test. Healthy and DR α-shape descriptors and FD measures were not normally distributed and therefore compared using Wilcoxon rank sum.

## Results

### Association of α-shape descriptors and FD with PDR

Images with PDR showed a significantly lower *OpA* when compared to Grade 0 (p = 0.001), Grade 1 (p = 0.01), Grade 2 (p = 0.02) and Grade 3 (p = 0.05), indicating a vasculature with less spread. A significantly lower *Grad*_*α*_ was observed in PDR when compared to Grade 0 (p = 0.002), Grade 2 (p = 0.02) and Grade 3 (p = 0.02) suggesting that PDR contained numerous small vessels. For *VS* there was no evidence of significant difference between groups following post-hoc testing and p-value adjustment. PDR exhibited a lower FD when compared to all other groups, indicating a vasculature that is less space filling (p < 0.001) (Table 1).

**Table 1:** Means and standard deviations of α-shape descriptors and FD parameter.

Parameter	Grade 0 (n = 547)	Grade 1 (n = 149)	Grade 2 (n = 239)	Grade 3 (n = 199)	PDR (n = 53)	Adjusted p-value	Post-hoc comparisons	Post-hoc p-value
*Opα*_*min*_, mean ± SD	45.2 ± 9.2	43.3 ± 8.4	45.5 ± 10.4	44.2 ± 9.9	43.3 ± 13.5	**.01**	Grade 0 vs PDR	**.05**
*OpA*, mean ± SD	443,652 ± 78,338	438,860 ± 73,985	432,539 ± 79,834	429,030 ± 82,898	400,363 ± 78,660	** < .001**	Grade 0 vs Grade 3 Grade 0 vs PDR Grade 1 vs PDR Grade 3 vs PDR	**.04** **.001** **.01** **.05**
*VS* mean ± SD	9,949 ± 1,476	10,286 ± 1,512	9,720 ± 1,735	9,864 ± 1,653	9,597 ± 1,857	**.03**	**-**	**-**
*Grad*_*α*_, mean ± SD	127 ± 22	132 ± 23	125 ± 27	125 ± 26	113 ± 26	** < .001**	Grade 0 vs PDR Grade 2 vs PDR Grade 3 vs PDR Grade 1 vs Grade 2 Grade 2 vs Grade 3	**.002** **.02** **.02** **.02** **.02**
FD, mean ± SD	1.44 ± 0.02	1.44 ± 0.02	1.43 ± 0.03	1.43 ± 0.03	1.42 ± 0.03	** < .001**	Grade 0 vs PDR Grade 1 vs PDR Grade 2 vs PDR Grade 3 vs PDR	** < .001** ** < .001** ** < .001** ** < .001**

P-values based on non-parametric ANOVA (K-Wallis) with correction for multiple comparisons (Benjamani-Hochberg).

*PDR* proliferative diabetic retinopathy, *n* number of images.

### Alpha-shape descriptors as features of PDR

Table 2 shows the number of times each feature reached a non-zero coefficient and average bootstrap performances. *Gradα* was the strongest feature for PDR classification. The feature that contributed the least for PDR classification was *Opα *_*min*_. Highest performances was achieved using all α-shape descriptors and FD.

**Table 2:** Relative feature importance.

Regulariser (λ)	*OpA*	*Opα *_*min*_	VS	*Grad*_*α*_	FD	AUC (95% CI)
Lasso (λ_min_)	**487**	360	356	**–**	**–**	0.67 (0.66 to 0.68)
Lasso (λ_1SE_)	**443**	174	212	**–**	**–**	
Elastic-net (λ_min_)	**451**	371	382	**–**	**–**	0.67 (0.66 to 0.68)
Elastic-net (λ_1SE_)	**451**	118	235	**–**	**–**	
Lasso (λ_min_)	441	402	451	**488**	**–**	0.73 (0.73 to 0.74)
Lasso (λ_1SE_)	315	190	272	**450**	**–**	
Elastic-net (λ_min_)	463	438	455	**491**	**–**	0.73 (0.73 to 0.74)
Elastic-net (λ_1SE_)	362	243	283	**451**	**–**	
Lasso (λ_min_)	385	377	445	441	**487**	0.76 (0.75 to 0.76)
Lasso (λ_1SE_)	202	187	316	**439**	259	
Elastic-net (λ_min_)	407	411	466	455	**494**	0.76 (0.75 to 0.77)
Elastic-net (λ_1SE_)	253	220	261	335	**460**	
Orlando et al. ^13^ (Lasso)	**–**	**–**	**–**	**–**	**√**	0.67 (0.52 to 0.80)

Relative feature importance calculated by the number of times a feature reached a non-zero coefficient per bootstrap, average bootstrap performances and performances reported in ^13^ using fractal dimensions to detect PDR. Bold indicates the most important feature.

*CI* confidence intervals, *AUC* area under curve.

### Comparison of alpha-shape descriptors and FD from manual annotations and automatic segmentations

Table 3 shows the mean relative errors for α-shape descriptors and FD with the manual segmentation as reference. Higher performances of the automatic segmentation were achieved in healthy individuals (DSC = 0.81870) compared to individuals with DR (DSC = 0.58530) demonstrating a high number of erroneous automatic segmentations in the DR group. Alpha-shape parameters exhibited mean relative errors between 10 and 43%, where a significant correlation was observed in *Grad*_*α*_ from Healthy individuals and FD from Healthy and DR. Greatest maximum relative error was observed in *OpA* measured from an individual with DR (manual *OpA* = 38; automatic *OpA* = 90; Max = 100%; DSC = 0.58528) who had large number of microaneurysms, haemorrhages and exudates that obstructed the vasculature and resulted in a sparse and fragmented segmentation. DR exhibited a higher *Opα*_*min*_*,* lower *OpA* (manual and automatic) compared to Healthy, indicating a vasculature that was less space filling, but was not significant between groups in measures derived from the manual or automatic segmentation. *Grad*_*α*_ was lower in DR compared to healthy individuals indicating the presence of small vessels but was not significant in measures derived from the manual or automatic segmentation. FD exhibited a 0.9% relative error in Healthy and a 1.4% error in DR individuals and was correlated to FD derived from manual segmentations. FD was significantly lower in Healthy (p = 0.01) and DR (p = 0.01), indicating a less space filling vasculature.

**Table 3:** The comparison of alpha-shape descriptors and FD obtained from manual annotation and automatic segmentation.

Parameter	Healthy (n = 15, DSC = 0.81870 ± 0.02360)			DR (n = 15, DSC = 0.58530 ± 0.0003)			DR vs Healthy (p-value*)	
	Manual	Automatic	MRE (max, p-value^+^)	Manual	Automatic	MRE (max, p-value^+^)	M	A
*Opα*_*min*_, mean ± SD	41.1 ± 5.6	52.4 ± 8.5	29% (80%, 0.476)	44.0 ± 9.4	58.0 ± 17.1	37% (100%, 0.235)	0.595	0.648
*OpA*, mean ± SD	1,063,768 ± 127,691	1,034, 863 ± 110,756	10% (24%, 0.144)	1,046,821 ± 88,366	1,004,089 ± 118,917	10% (26%, 0.281)	0.367	0.539
*VS*, mean ± SD	16,062 ± 3182	20,021 ± 2464	22% (39%, 0.087)	24,516 ± 4270	18,113 ± 3,379	26% (49%, **0.008**)	0.305	0.202
*Grad*_*α*_, mean ± SD	366 ± 54	250 ± 57	31% (55%, **0.005**)	362 ± 89	213 ± 62	43% (66%, 0.064)	0.967	0.249
FD, mean ± SD	1.47 ± 0.01	1.46 ± 0.01	0.9% (2%, **0.002**)	1.47 ± 0.01	1.45 ± 0.01	1.4% (3%, **0.004**)	**0.01**	**0.01**

*DSC* dice similarity coefficient with manual annotation as reference, *MRE* mean of the relative error with manual annotation as reference, *Max* maximum relative error with manual annotation as reference. n, number of images.

^+^Pearson correlation test with null hypothesis that the correlation coefficient is zero.

*Wilcoxon rank sum with null hypothesis that medians are equal between groups.

## Discussion

We implemented α-shapes as a method to quantify retinal microvasculature morphology from fundus imaging and investigated its application to the task of PDR characterisation. A significantly lower FD was observed in PDR compared to all other DR grades suggesting that the vasculature is less space filling in PDR. We did not observe the increase in FD of PDR eyes as reported in^13^, likely due to the removal of duplicates, alteration of DR grades (as specified on the MESSIDOR project website) and parameter choice (e.g. random sampling on the vessel map), highlighting a challenge with the repeatability of FD analysis. The α-shape from which the features proposed here are extracted would not be affected by parameter choice as no random sampling is conducted and there is only one α-shape for a point set^20^.

We experimented with *Opα*_*min*_ as a measure of complexity and *OpA* as a measure of vessel spread and observed that PDR had a significantly lower *OpA* compared to all other DR grades and a lower *Opα*_*min*_ compared to grade 0. This suggests that this the vasculature was dense (i.e. the vessels occupy a smaller area and consists of many ‘parts’). *OpA* was determined to be the most distinguishable feature of PDR and was the most important feature for PDR classification for lasso and elastic-net (when *Grad*_*α*_ and FD were not included as features for classification), suggesting that a decreased vessel spread could translate to a predictive marker of progression to PDR.

When *Grad*_*α*_ was introduced into the model (without FD) this became the most important feature. PDR had a lower mean *Grad*_*α*_ compared to all other grades suggesting the vasculature contains numerous small vessels indicating the presence of neovascularisation. However, the limitation to this parameter is that the presence of numerous small erroneous segmentations of PDR related pathology (e.g., haemorrhages that exhibit the same imaging characteristics as vessels and confounds the automatic segmentation process) could be contributing to the lower *Grad*_*α*_ observed. Indeed, *Grad*_*α*_ was significantly lower in PDR when comparing to grade 0, 2 and 3 but was not observed in grade 1. Individuals with grade 1 DR contain a small number of microaneurysms and is therefore expected to be vastly different from PDR. Grade 1 was the second smallest group in the MESSIDOR dataset and may be underpowered to detect a significant difference between PDR and grade 1. However, assuming that the dataset was sufficiently powered other reasons could include image quality such as the presence of artefact that may be contributing to a similar distribution of *Gradα* measured from PDR cases. To address such limitations, manual correction of the vessel segmentation or improvements to automatic vessel segmentation would be required. Nevertheless, *Grad*_*α*_ could be a potential metric to evaluate segmentation quality in datasets that are several orders of magnitude greater than MESSIDOR where visual assessment and manual correction of the vessel skeleton prior to quantification of morphology is not feasible.

We achieved good classification performance for PDR (AUC 0.67 to 0.76) that was improved with the addition of FD as a feature into the model and was comparable to the performance achieved by Orlando et al.^13^. Orlando et al. further improved their performance to AUC 0.91 when red lesion features were included as features for PDR classification^13^. We used fivefold cross validation and bootstrap to obtain performance measures and feature importance, when larger PDR datasets become available a held out experimental set-up should be implemented along with the addition of red lesion features if available.

We conducted a comparison of α-shape descriptors and FD measured from manual and automatic segmentations to assess measurement accuracy. The main differences between the manual and automatic segmentations are the presence or absence of small vessels. Additionally, manual annotations in the HRF are meticulously drawn, such that vessels are annotated to the complete length of the path. As vessels taper towards the end, they can lose contrast and may not be detected by the algorithm. In previous work that compared FD measured from automatic segmentations to manual annotations from two human observers (DRIVE dataset) reported mean relative errors between 7.1 and 9.3% and attributed this to the accuracy of the segmentation method^17^. We showed that α-shape parameters are also affected by segmentation accuracy. We therefore recommend careful selection of segmentation algorithms (e.g., use of multiple algorithms to assess reproducibility) to ensure α-shape and FD measures are as reliable as possible. Experimental results did not show a significant difference between healthy and DR that could be due to low sample size and presence of a broad range of DR severities in the DR group. FD measures obtained from manual and automatic segmentations showed a significant difference between groups, suggesting that it may be more sensitive than α-shape descriptors.

Interpretation of objective measures derived from the automatic segmentation of the retinal vasculature requires assessment of statistical significance of difference between groups and their clinical importance. To establish a threshold that identifies a change to be clinically important requires group-level between patient contrasts and within-patient longitudinal contrasts and therefore could not be established here. Nevertheless, providing careful segmentation procedures are followed, α-shapes appear to be a promising measure of vascular geometry. Future applications of α-shapes for describing retinal vasculature morphology could be applied to research involving the assessment of vascular changes in other ocular diseases as well as characterising vascular changes related to systemic comorbidities or neurodegenerative diseases that are increasingly reported to manifest in the retina^33,34^.

## Data Availability

The datasets generated during and/or analysed are available from the author on reasonable request.
